# Mandibular metastasis from carcinoma of the bladder: Report of a case and literature review

**DOI:** 10.4317/jced.57293

**Published:** 2022-04-01

**Authors:** Ilaria Giovannacci, Paolo Vescovi, Luigi Corcione, Domenico Corradi, Ronell Bologna-Molina, Marco Meleti

**Affiliations:** 1 DDS, Msc. Clinical and Experimental Medicine PhD Program, University of Modena and Reggio Emilia, Modena, Italy; 2 DDS, Msc, PhD. Centro Universitario di Odontoiatria, Department of Medicine and Surgery, Oral Medicine and Laser Surgery Unit, University of Parma, Parma, Italy; 3 MD. Department of Medicine and Surgery, Section of Human Pathology and Histopathology, University of Parma, Parma, Italy; 4 DDS, PhD. Molecular Pathology Area, School of Dentistry, Universidad de la República, Uruguay; 5 DDS, PhD. Centro Universitario di Odontoiatria, Department of Medicine and Surgery, Oral Medicine and Laser Surgery Unit, University of Parma, Parma, Italy

## Abstract

Metastases represent about 1% of all malignant tumors of the oral region. Only 12 cases of metastases to the jawbones and 3 to the oral soft tissues from a carcinoma of the bladder are reported in the English literature. Here we report a case of an 86 year-old man with a metastasis to the anterior region of the lower jaw from a transitional cell carcinoma of the bladder treated 5 years before, all-together with a literature review.

** Key words:**Bladder, mandibular metastasis, oral metastasis, transitional cell carcinoma.

## Introduction

The oral cavity is an uncommon site of metastatic localization from solid tumors. Oral metastases (OMs) represent about 1% of all malignant tumors at this site. In about 25% of cases, OMs are the first manifestation of the spreading of malignant cells at a distant site and in 23% of cases of OMs have led to the diagnosis of an occult primary (1).

The jawbones, particularly the posterior region of the mandible (82% of cases), are more frequently affected than the soft tissues (hard to soft tissues ratio = 2:1). The gingiva is the most commonly involved soft tissue site (54%) (1).

In male patients, solid primary tumors that can metastasize to the jawbones affect the lungs, prostate, kidneys and liver; in women, OMs mainly derive from breast, adrenal glands, female genital organs and colorectal tumors.

The most common histologic type of malignancy of the urinary bladder is the transitional cell carcinoma (TCC). Most TCCs are locally aggressive neoplasms that can eventually metastasize.

Metastases to the head and neck region are very rare, the most frequently affected sites being the brain and the supraclavicular neck nodes (2).

To the best of our knowledge, 15 cases of OMs from TCC of the urinary bladder have been reported (2-7).

Here we report a case of metastasis to the oral cavity from a TCC of the bladder diagnosed and treated 5 years before, altogether with a literature review.

## Case Report

The present research has been conducted in accordance to the Ethical Guidelines of the Academic Hospital of Parma. A written consensus was obtained by the patient described in the case report.

**Figure 1:**
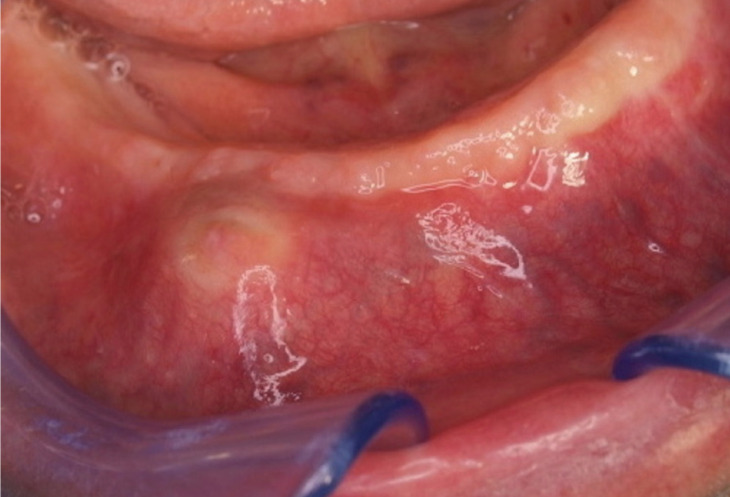
Intraoral view showing a vestibular swelling of the right edentulous mandible.

An 86 year-old man with a compromised medical situation was referred to the Center of Oral Laser Surgery and Oral Medicine, University of Parma, Italy, because of a swelling in the right anterior region of the edentulous mandible (Fig. 1).

The lesion was present since one month. The patient reported that homolateral lower lip paresthesia was present since the last three weeks.

Anamnesis disclosed a history of TCC of the bladder that was supposedly radically excised 5 years before. Other findings included an acute myocardial infarction and a recent pathologic fracture of the head of the femur. Such a fracture was surgically reduced without success.

**Figure 2:**
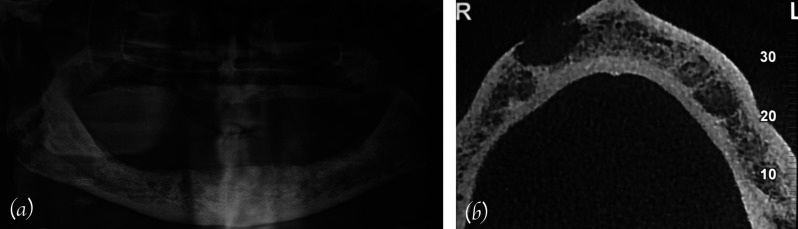
a) OPT showing no specific signs of bone alterations; b) CT scan showing mandibular destruction around the mental foramen.

No specific signs of bone alterations could be detected on orthopathomography (OPT) (Fig. 2a). Nevertheless, computed tomography (CT) scan showed a grossly rounded osteolytic area of about 1.5 cm in diameter in the spongious bone with destruction of the vestibular plate (Fig. 2b). The osteolysis was localized around the mental foramen.

Mainly on the basis of the anterior localization, working diagnosis included a focal lesion of a multiple myeloma, lymphoma and osteosarcoma.

An incisional biopsy in the central part of the lesion was performed.

**Figure 3:**
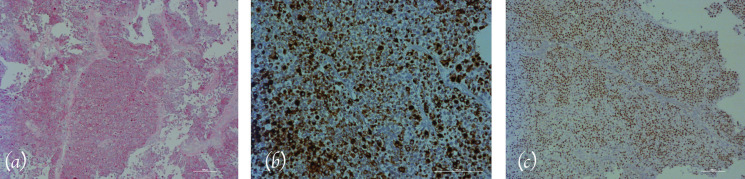
a) Histopathologic view showing a very high cellularity (Hematoxillin-eosina, H&E stain-ing); b) Immunohistochemical staining of the oral lesion (Cytokeratin 20, original magnifi-cation: 20X); c) Immunohistochemical staining of the oral lesion (Protein 63, original magnification: 10X).

Surprisingly, the histopathological examination demonstrated the presence of a carcino-ma deriving from the urothelium (Fig. 3a). In particular, immunohistichemical stains marked a strong positivity for cytokeratin 20 (which is positive, among other tissues in gastrointestinal epithelium and urothelium) and for protein 63 (p63) (a nuclear staining posive, among others in proliferating epithelia from the uterine cervix, urothelium and prostate) (Fig. 3b,c). Focal positivity for cytokeratin 14 (stratified epithelia) and for cytokeratin 7 (transitional epithelia) was also present.

A histopathologic diagnosis of metastasis, most likely from an unknown recurrence of the TCC of the bladder was rendered.

Further work-up (CT scan total body, and positron emission tomography - PET scan) showed the presence of a neoplastic process of the bladder altogether with tracer up taking lesions localized at the head of the femur and in the mandible.

The patient was referred to the oncology department and he died for the disease 6 months later.

## Discussion

Transitional cell carcinoma (TCC) is the most common histological type of bladder cancer (approximately 97% of malignant tumors of the bladder) (7).

The most common sites of metastases from TCC are lungs, liver, brain and bones. In the head and neck region, sites of metastases localization include the supraclavicular nodes and the skull (2,7).

The metastatic colonization of the oral tissues is exceedingly rare. To the best of our knowledge only 12 cases of metastases to the jawbones and 5 to the oral soft tissues, without bone involvement, have been reported in the English literature (2-9) ( Table 1) 

**Table 1:**
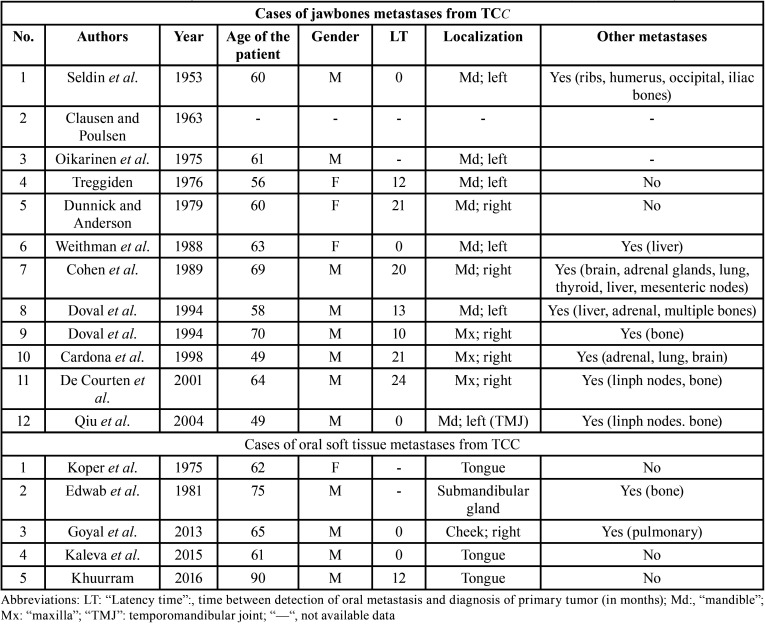
Cases of metastases to the jawbones and to the oral soft tissues from transi-tional cell carcinoma (TCC) of the urinary bladder.

A higher prevalence of metastases from TCC among men is evident (75% of cases in men; M/F: 2.75:1). Therefore, Laor *et al*. hypothesized a minor aggressiveness of bladder TCC in female patients (10). It is opinion of the authors that such a discrepancy may in-stead depend on the higher prevalence of TCC of the bladder in men, rather than on the higher aggressiveness of this tumour in males. In fact, the prevalence of carcinoma of the urinary bladder is more predominant in male (88.7%) than female (11.3%) (11).

The majority of metastatic TCCs to the oral region are diagnosed in patients in the sixth decade (mean age: 63 years; ranging from 49 to 90 years).

Among cases of OMs localized in the hard tissues, 8 were localized in the mandible (66.7%), 3 in the maxilla (25%) and 1 in temporomandibular joint (8.3%). Metastases from TCC to oral soft tissue were localized in the tongue (60%), cheek mucosa and in the submandibular gland (2-9).

The bone marrow seems to be a target site for metastatic cells. Jaw metastases usually occur distally to the canine area, where presence of bone marrow is higher. Hirshberg *et al*. speculated that the thin-walled sinusoidal channels of the marrow might be a good site for settlement and proliferation of malignant cells. Add to this, the growth factors in the marrow could stimulate the metastasis growth (1,2).

In patients with malignant tumors, a metastasis to the oral cavity can well be included within the range of possible diagnoses when a clinically suspect lesion occurs at this site. However, up to 23% of patients with OMs have an undiagnosed primary (1). In case of diagnosis of OM from an unknown tumor, medical investigations should include a detailed anamnesis, physical examination including pelvic and rectal exploration, complete blood count, comprehensive metabolic and electrolyte studies, urinalysis, occult faecal blood tests and a chest radiograph. Further work up should include CT scans of the abdomen and pelvis for possible primaries of the pancreas, liver, adrenal glands, kidneys, prostate, bladder, ovaries and stomach. PET scanning with fluorodeoxyglucose (FDG), particularly when other diagnostic tools fail to identify a source for malignant cells, may provide some help (12).

When OMs are localized to the jawbones, the most common clinical manifestation is a rapidly growing swelling associated to pain and mobility of adjacent teeth. Other signs and symptoms may be present, depending on the localization of the lesion: trismus if OMs are located in the condyle; symptoms related to the sinus (e.g. nasal occlusion, rhinorrea, nosebleed and cephalgy) and exophthalmos in maxillary lesions; mental nerve neuropathy (‘‘numb chin syndrome’’) in mandibular metastatic localization (13).

OM should be suspected also when a post-extractive socket does not heal in an adequate time.

With regard to the localization in soft tissues, the clinical presentation is a sub-mucosal mass of soft consistence, fixed on the underlying tissue and possibly ulcerated and bleeding lesions. At gingival level these lesions are usually exophytic, having an epulis-like appearance.

Clinically, OMs can be misdiagnosed for other primary oral lesions mainly because of their rarity. Differential diagnoses include malignant lesions such as primary squamous cell carcinoma, salivary gland tumors, lymphomas and benign locally aggressive entity such as giant-cell granuloma (7).

Radiographic features of the majority of OMs include a lytic radiolucent appearance, mostly ill-defined, sometimes with a multiloculated or multilobulated pattern. Occasionally radiopaque focal areas, most probably depending on new bone formation, can be observed (17% of OMs) (13). The incidence of sclerotic changes is low, 10 % of the cases, most originating from prostatic carcinomas, followed by breast and thyroid carcinomas (14).

In approximately 5 % of the cases, conventional radiographic examination (e.g. OPT, intraoral radiographies), do not show any pathological changes (1).

An OM from genitourinary malignancies is usually the epiphenomenon of a widespread metastatic disease. In fact, only 2 out of 13 patients (15.39%) with OM from primary TCC had no further metastases.

## Conclusions

Metastatic tumors of the oral cavity (both in hard and soft tissues) are uncommon and are the evidence of a wide spread disease. The diagnosis of a metastatic lesion in the oral region is challenging both in recognition as metastatic lesion and in determination of the site of origin, but but it is essential to identify them early because OMs are considered a very negative prognostic factor being associated to a 1-year survival rate of about 0% after the diagnosis.
